# Correlation of gene expression with magnetic resonance imaging features of retinoblastoma: a multi-center radiogenomics validation study

**DOI:** 10.1007/s00330-023-10054-y

**Published:** 2023-08-24

**Authors:** Robin W. Jansen, Khashayar Roohollahi, Ogul E. Uner, Yvonne de Jong, Christiaan M. de Bloeme, Sophia Göricke, Selma Sirin, Philippe Maeder, Paolo Galluzzi, Hervé J. Brisse, Liesbeth Cardoen, Jonas A. Castelijns, Paul van der Valk, Annette C. Moll, Hans Grossniklaus, G. Baker Hubbard, Marcus C. de Jong, Josephine Dorsman, Pim de Graaf, On behalf of the European Retinoblastoma Imaging Collaboration

**Affiliations:** 1 Department of Radiology and Nuclear Medicine, Amsterdam UMC, Vrije Universiteit Amsterdam, De Boelelaan 1117, 1081 HV Amsterdam, The Netherlands; 2 Cancer Center Amsterdam, Amsterdam, The Netherlands; 3 Department of Oncogenetics, Amsterdam UMC, Vrije Universiteit Amsterdam, Amsterdam, The Netherlands; 4 Department of Ophthalmology, Casey Eye Institute, Oregon Health & Science University, Portland, USA; 5 Emory Eye Center, Ocular Oncology Service, Atlanta, USA; 6 Department of Ophthalmology, Amsterdam UMC, Vrije Universiteit Amsterdam, Amsterdam, The Netherlands; 7 Institute of Diagnostic and Interventional Radiology and Neuroradiology, University Hospital Essen, Essen, Germany; 8 Department of Diagnostic Imaging, University Children’s Hospital Zurich, University of Zurich, Zurich, Switzerland; 9 Department of Radiology, Centre Hospitalier Universitaire Vaudois, Lausanne, Switzerland; 10 Azienda Ospedaliera Universitaria Senese, Siena, Italy; 11 Imaging Department, Institut Curie Paris, Paris, France; 12 Department of Pathology, Amsterdam UMC, Vrije Universiteit Amsterdam, Amsterdam, The Netherlands

**Keywords:** MRI, Gene expression, Radiogenomics, Retinoblastoma, Validation

## Abstract

**Objectives:**

To validate associations between MRI features and gene expression profiles in retinoblastoma, thereby evaluating the repeatability of radiogenomics in retinoblastoma.

**Methods:**

In this retrospective multicenter cohort study, retinoblastoma patients with gene expression data and MRI were included. MRI features (scored blinded for clinical data) and matched genome-wide gene expression data were used to perform radiogenomic analysis. Expression data from each center were first separately processed and analyzed. The end product normalized expression values from different sites were subsequently merged by their *Z*-score to permit cross-sites validation analysis. The MRI features were non-parametrically correlated with expression of photoreceptorness (radiogenomic analysis), a gene expression signature informing on disease progression. Outcomes were compared to outcomes in a previous described cohort.

**Results:**

Thirty-six retinoblastoma patients were included, 15 were female (42%), and mean age was 24 (SD 18) months. Similar to the prior evaluation, this validation study showed that low photoreceptorness gene expression was associated with advanced stage imaging features. Validated imaging features associated with low photoreceptorness were multifocality, a tumor encompassing the entire retina or entire globe, and a diffuse growth pattern (all *p* < 0.05). There were a number of radiogenomic associations that were also not validated.

**Conclusions:**

A part of the radiogenomic associations could not be validated, underlining the importance of validation studies. Nevertheless, cross-center validation of imaging features associated with photoreceptorness gene expression highlighted the capability radiogenomics to non-invasively inform on molecular subtypes in retinoblastoma.

**Clinical relevance statement:**

Radiogenomics may serve as a surrogate for molecular subtyping based on histopathology material in an era of eye-sparing retinoblastoma treatment strategies.

**Key Points:**

*• Since retinoblastoma is increasingly treated using eye-sparing methods, MRI features informing on molecular subtypes that do not rely on histopathology material are important.*

*• A part of the associations between retinoblastoma MRI features and gene expression profiles (radiogenomics) were validated.*

*• Radiogenomics could be a non-invasive technique providing information on the molecular make-up of retinoblastoma.*

**Supplementary information:**

The online version contains supplementary material available at 10.1007/s00330-023-10054-y.

## Introduction

Retinoblastoma is the most common primary intraocular cancer in children, occurring in one in every 15,000–20,000 live births [1]. It is also one of the few malignancies that is routinely treated without prior histopathologic or genetic analysis. Recently developed treatments such as selective intra-arterial chemotherapy (SIAC) and intravitreal chemotherapy have expanded the area of eye-sparing treatments, being increasingly used for more advanced retinoblastoma [2–5]. In making treatment decisions, clinicians must rely on ophthalmologic assessment and imaging instead of information derived from fresh tumor tissue. In an era where oncologic treatments are increasingly tailored to specific genetic traits, retinoblastoma poses a challenge given no such information is available in clinical practice. Therefore, non-invasive methods informing on molecular tumor make-up are vital for the development of tailored therapies in retinoblastoma.

An emerging non-invasive method informing on molecular traits is radiogenomics, in which association between imaging features and molecular features are evaluated. Recently, whole-genome gene-expression profiles were compared with magnetic resonance imaging (MRI) in retinoblastoma [6]. A predefined photoreceptorness gene-expression signature was evaluated because loss of this photoreceptorness gene expression is associated with tumor progression and distinctive ex vivo chemotherapy susceptibility [7, 8]. In the initial study, photoreceptorness loss was associated with advanced stage imaging features such as a large number of tumors and tumors located in the entire retina or the entire globe. However, radiogenomic results were not validated yet. Lack of validation studies is, however, a major impediment for further progress towards clinical applicability of radiogenomics. In a recent systematic review evaluating radiogenomic studies, 88% (166/188) of studies did not perform validation in an independent cohort [9]. However, there is general consensus that validation studies are vital in this new field of research [10–15]. The rare nature of retinoblastoma translates to paucity of datasets of patients with MRI and gene expression profiles, which will become increasingly scarce with the rise of eye-sparing treatments. Thus, timely validation of radiogenomic findings is pressing. By elucidating relationships between imaging features and gene expression profiles, this study may aid in risk stratification and selection for targeted treatment.

The purpose of this study was to assess the repeatability of previously found associations between imaging features and gene expression profiles. The main focus was on validation of associations between imaging features and the pre-defined gene-expression signature of photoreceptorness [7, 8].

## Materials and methods

The Institutional Review Board of the VU University Medical Center (IRB00002991) in Amsterdam, The Netherlands, approved this multicenter retrospective study with waiver of informed consent.

### Patient selection

MRI and gene expression data were collected from (1) Emory Eye Center, Atlanta, Georgia, USA; (2) Amsterdam UMC, location VUmc, the Netherlands; and (3) Essen University Hospital, Essen, Germany. Patients were included if there was (1) histopathologic diagnosis of retinoblastoma; (2) pre-treatment MRI available including at least T1-, T2-, and T1 contrast-enhanced sequences; and (3) genome-wide gene expression data. The extreme outliers for the expression studies that drastically diverged from their corresponding cohort were excluded, as these may signify low mRNA profiling quality and/or a high percentage of non-tumor material. The hierarchical clustering analysis with WARD2 method and based on the normalized expression values was performed. Subsequently, samples that widely branched out main clusters and sub-branches of the cohorts were removed from the analysis. In total, 2 samples from Amsterdam and 4 samples from Atlanta were designated as outliers by their clustering patterns and subsequently discarded. Seventeen patients were previously reported in a study on genetic markers for high-risk retinoblastoma [16], while the current study reports on associations between MRI features and gene-expression profiles.

### Magnetic resonance imaging assessment

A previously validated imaging atlas with definitions and examples of features was adopted for scoring [6]. This atlas was composed by radiologists with experience in retinoblastoma MRI from the European Retinoblastoma Imaging Collaboration and compiled 25 MRI features that may be encountered in retinoblastoma imaging. The imaging features that were assessed included features informing on tumor morphology (e.g., shape, definition of tumor margin), intra-tumoral findings (e.g., calcifications, proportion of enhancement), and intra-ocular findings (e.g., number of tumor lesions, vitreous seeding). Two radiologists with expertise in retinoblastoma imaging (M.C.d.J. and P.d.G., with 10 and 17 years of experience, respectively) individually scored the features blinded for patient data. Inter-reader agreement was calculated using Cohen’s kappa. Subsequently, discrepancies were resolved by consensus and the consensus scores were adopted for association with gene expression values. The MRI units included 1.5-T (*n* = 19, 53%) and 3-T (*n* = 17, 47%) systems, with an average pixel size of 0.34 mm (range 0.27–0.90 mm), an average section thickness of 0.90 mm (range 0.6–3 mm), and an average intersection gap of 0.75 mm (range 0.3–3.6 mm).

### Gene expression data processing and analysis

To avoid non-biological variations caused by differences in platforms and procedures, the expression data from each referral center were first separately processed and analyzed. The end products of each analysis were merged via adapting scaling adjustment (i.e., *Z*-score), enabling cross-center downstream analysis.

#### Amsterdam data

Gene expression data were obtained by whole-genome total RNA sequencing. Total RNA was extracted from 16 primary retinoblastoma samples by dual RNA/DNA isolation protocol from tissue frozen in OCT (AllPrep DNA/RNA—QIAGEN). Library preparation was carried out using SMARTer Stranded Total RNA-Seq kit v3 (Takara bio group). The RNA samples were paired-end sequenced by Novaseq6000 at 60 × coverage and 100-bp fragment sizes. The FASTQ files were cleaned by fastp [17]. Sequence files were mapped to the human reference genome 38 (hg38) by HISAT2 [18]. BAM conversion, sorting, and indexing were done by SAMTOOLS [19]. The feature count was utilized in counting gene-level reads [20]. Multi-mapping reads were incorporated by fractional counting. The expression analysis was performed using edgeR [21]. Original library size was normalized into effective library size by the trimmed mean of *M*-value (TMM). Significant testing was performed by fitting the generalized linear model (GLM). Differential expressions levels with FDR < 0.05 were considered significant.

#### Atlanta and Essen data

The transcriptomic data were profiled by microarray experiments using Affymetrix pd.hugene.2.0.st and hgu133a arrays respectively. CEL files from Atlanta and Essen were read into expression objects using oligo and Affy packages respectively [22, 23]. Data were normalized by robust multi-chip average (RMA) using the Limma package [24]. Due to array type differences between the sites, data were separately normalized. The differential expression analysis was done by fitting GLM. Multiple hypothesis testing was performed based on the Benjamini and Hochberg false discovery rate adjustment method, considering *p* < 0.05 significant.

### MRI–photoreceptorness correlation analysis

Within each gene expression platform, the photoreceptorness score per sample was calculated as the averaged normalized expression of the predefined list of 2753 photoreceptorness genes [7]. The photoreceptorness scores were scaled by calculating the* Z*-score. Approximate sample photoreceptorness relative to the mean* Z*-scores from study sites were combined and associated with patient-matched MRI feature scores using Spearman’s rank correlation coefficient or the Kruskal–Wallis test in accordance with the test study, unless when lack of data points did not permit association analysis (i.e., less than 3 alternative scores per feature). Correlation analysis was performed within R, considering *p* < 0.05 significant.

### Validation of differentially expressed genes within MRI feature groups

The contrast design between the test and validation studies was not comparable, impeding repeated analysis with a relatively comparable design matrix and statistical power. To address the issue, two analyses were performed. Firstly, sample relationships were examined by the differentially expressed genes within MRI feature in the previous study (test): number of lesions (*n* = 70 genes), tumor location (*n* = 355 genes), subretinal seeding (*n* = 873 genes), and vitreous seeding (*n* = 19 genes). The normalized expressions of the genes previously identified as differentially expressed for features were extracted in the current sample (validation set). This was followed by performing *k*-mean clustering (*k* = 3) and principal component analysis (PCA). The partitioning outcomes and the first two principal components of expression were then overlaid and plotted to examine the relationship between samples with different MRI feature scores.

Secondly, the differential expression analysis within each site was performed when at least three samples at each side of the contrast design were available, permitting a minimum reliable statistical power. Subsequently, the end results of the analysis were merged, and gene ontology enrichment analysis was performed by Toppgene (https://toppgene.cchmc.org/).

## Results

### Patients and MRI assessments

Thirty-six retinoblastoma patients from three retinoblastoma referral centers in Atlanta, USA (*n* = 17), Amsterdam, Netherlands (*n* = 13), and Essen, Germany (*n* = 6) were included in this study. Of the patients, 14 were female (42%), 34 (94%) were unilateral, mean age at scan date was 24 months (SD 18 months), and mean MRI examination year was 2012 (SD 6 years, range 2002–2018). The findings within this set (hereafter referred to as “validation set”) were compared with the analysis of the “test set” for which patient demographics were previously described [6]. Cohen’s kappa for inter-reader agreement for adopted imaging features was on average 0.42 (moderate agreement), ranging from 0 to 0.68 (Appendix Table 1).

### Radiogenomics validation: imaging features’ association with photoreceptorness gene expression score

**Fig. 1:**
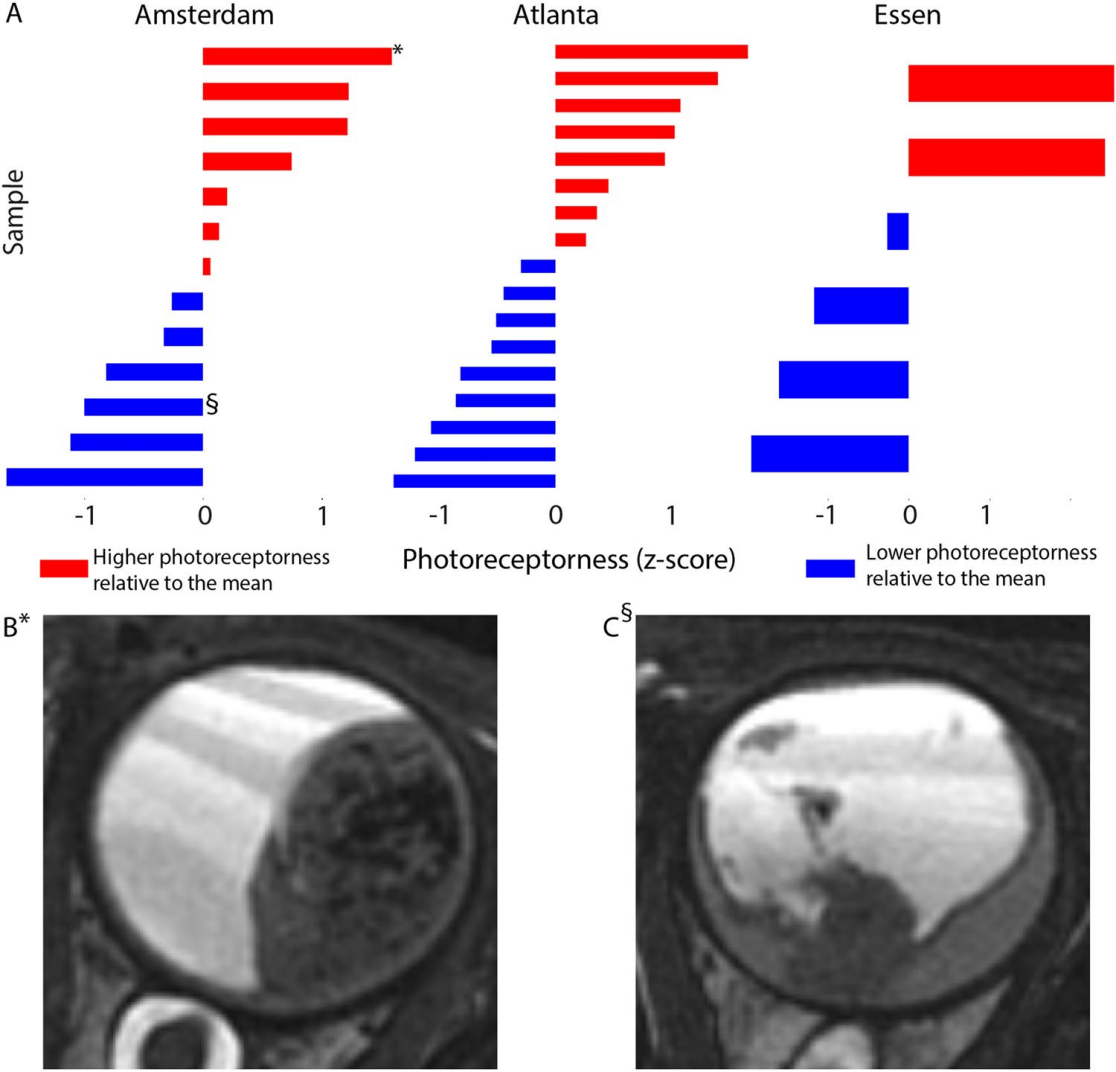
**A** *Z*-scores for photoreceptorness gene expression score in retinoblastoma among different centers. **B** MR imaging example of high photoreceptorness retinoblastoma (case*). **C** MR imaging example of low photoreceptorness retinoblastoma (case §)

**Fig. 2:**
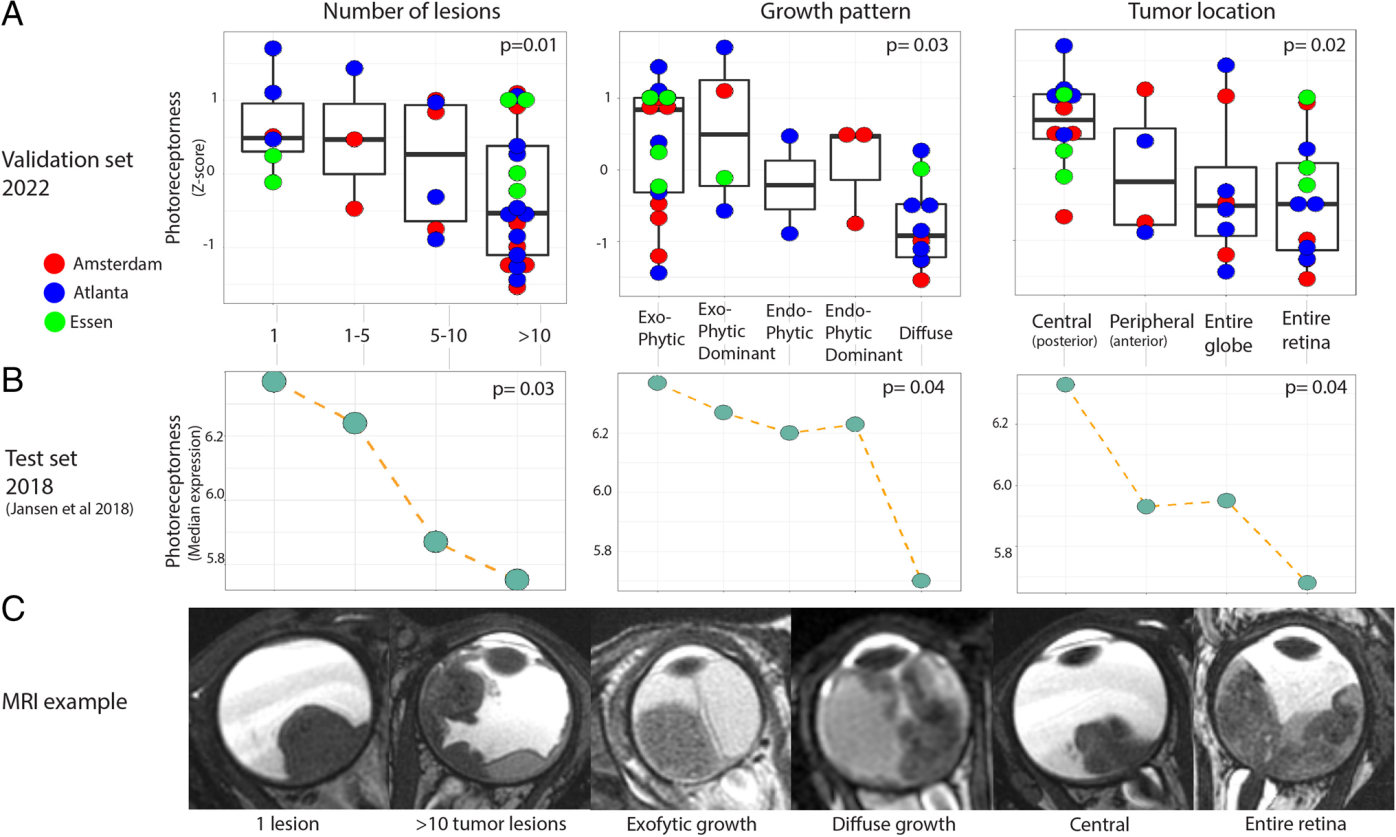
Non-parametric correlation analysis between photoreceptorness gene expression and MRI imaging features. Number of lesions, growth pattern, and tumor location were validated to be associated with the photoreceptorness gene expression profile. **A** Boxplots indicating the overall photoreceptorness per MRI feature/score. Points present the photoreceptorness score of individual samples, presented as a *z*-score. The point colors are customized based on corresponding referral centers of the tumors. In line with the test study, the number of lesions negatively correlates with photoreceptorness score. Tumors with diffuse growth pattern show an overall decreased photoreceptorness, as did tumors encompassing the entire globe or the entire retina. **B** Dot plots illustrating the median photoreceptorness expression per MRI feature from the test study as comparison. **C** MRI images featuring examples of different MRI characteristics represented in the plots of panels **A** and **B**

**Fig. 3:**
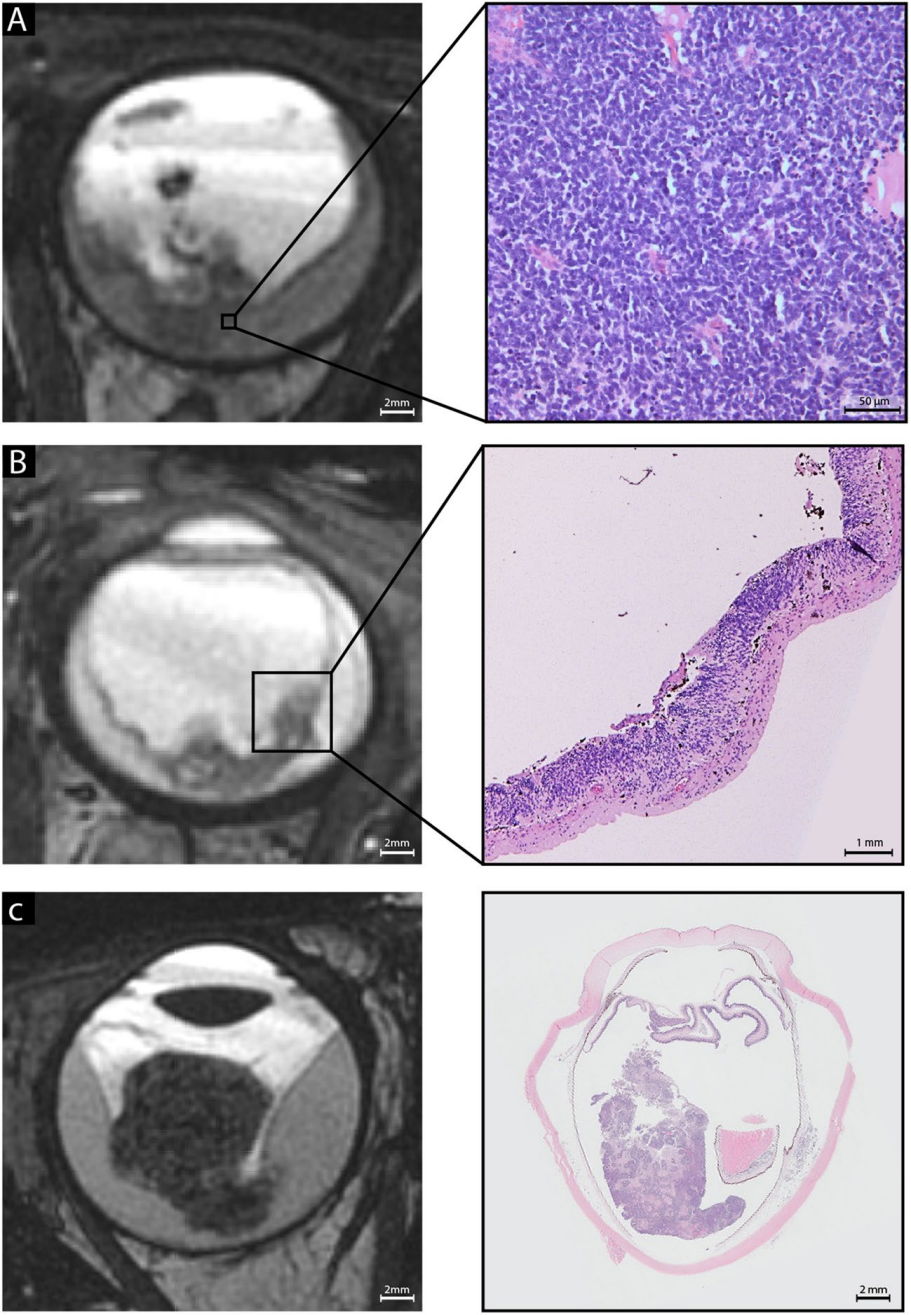
Radiological-pathological correlations showing T2-weighted MR imaging features that were validated to be associated with low photoreceptorness, a gene expression profile whose low expression implies tumor progression. Imaging and pathological features suggest advanced disease progression and poor differentiation. **A** A large amount of tumors on MR imaging and corresponding tumor histopathology. The high magnification histopathology allows for displaying the absence of rosettes, in particular Flexner–Wintersteiner rosettes, implying poor differentiation. High pathology magnification shows absence of rosettes. **B** Radiologically diffuse tumor growth within the retina with histopathological loss of normal layer architecture. **C** A tumor location in the majority of the retina/globe on both MR imaging and histopathology. For imaging examples of antagonist features associated with high photoreceptorness (a small amount of lesions, exofytic growth, central location), see Fig. 2, panel C

Similar to the findings in the test set, the photoreceptorness gene expression score showed a gradual distribution among samples from the three centers; examples of a low and a high photoreceptorness case were provided (Fig. 1). Three MRI features that significantly correlated to the photoreceptorness gene expression score in the test set were also found to be correlated with photoreceptorness in the current validation set: number of lesions (multiple lesions showing lower photoreceptorness, *p* = 0.01), growth pattern (diffuse growth showing lower photoreceptorness,* p* = 0.03), and tumor location (tumor in the entire globe or entire retina showing lower photoreceptorness, *p* = 0.02) (Table 1). For these MRI features, the differential expression of photoreceptorness was remarkably similar for the feature categories (Fig. 2) in the validation set compared with the test set. The number of lesions showed an inverse linear relationship with photoreceptorness in the validation set, with multifocality showing low photoreceptorness similar to findings from the test set. Similarly, diffuse tumor growth was again found to be correlated with low photoreceptorness. In both the test set and the validation set, tumors within the entire retina or filling the entire globe showed low photoreceptorness expression, while tumors with greater components behind the equator exhibited higher photoreceptorness expression. Therefore, the validated MRI phenotype of low photoreceptorness retinoblastoma consisted of multifocal, diffuse-growing retinoblastoma filling the globe or growing along the entire retina (Fig. 2). For this imaging phenotype, combined radiology and histopathology figures were presented for low photoreceptorness cases (Fig. 3). Histopathology assessment of the three cases with the lowest photoreceptorness from the Amsterdam center showed lack of rosettes (particularly Flexner-Wintersteiner rosettes), indicating poor differentiation (Fig. 3) [25, 26]. Other imaging-photoreceptorness correlations from the test set were not validated: eye size and increased choroidal enhancement beneath the tumor were significantly associated with photoreceptorness in the test set, but not in the validation set. Statistically significant findings associated with lower photoreceptorness only in the current validation set included plaque-shaped tumors (*p* = 0.01) and tumors with irregular margins (*p* = 0.01) (Appendix Fig. 1).

**Table 1:** Validation of associations of imaging features with photoreceptorness gene expression signature

MRI feature	Validation **set** Statistical difference in photoreceptorness gene signature (*p-*values)	Test set (Jansen et al 2018) Statistical difference in photoreceptorness gene signature (*p-*values)
Number of lesions (multiple lesions showing lower photoreceptorness)	0.01*	0.03*
Growth pattern (diffuse growth showing lower photoreceptorness)	**0.03^**	**0.04^**
Tumor location (tumor in the entire globe or retina showing lower photoreceptorness)	**0.02^**	**0.04^**
Eye size	0.40*	< 0.001*
Enhancement choroid beneath tumor	0.16^	**0.03^**
Dominating shape of largest tumor lesion (plaque-shaped tumors showed lower photoreceptorness)	**0.01^**	0.18**^**
Irregular/ill-defined tumor margins	0.01*	0.20*
Calcifications	0.71*	0.19*
Compactness of the entire mass	0.27*	0.77*
Enhancement anterior eye segment	0.42*	0.07*
Proportion contrast-enhancing tumor (CET)	0.63*	0.58*
Proportion necrosis	0.63*	0.58*
Retinal detachment	0.24*	0.98*
Shallowness of the anterior eye chamber	0.70^	0.42^
Subretinal composition	0.60^	0.11^
Subretinal seeding	0.64*	0.06*
Tumor homogeneity	0.12^	0.56^
Vitreous hemorrhage	0.20^	0.52^
Vitreous seeding	0.36*	0.10*

Data in bold are validated: statistical difference in both test and validation sets. *p*-values derived from *Spearman or ^Kruskal Wallis tests

### Radiogenomics validation: differentially expressed genes for MRI features

**Fig. 4:**
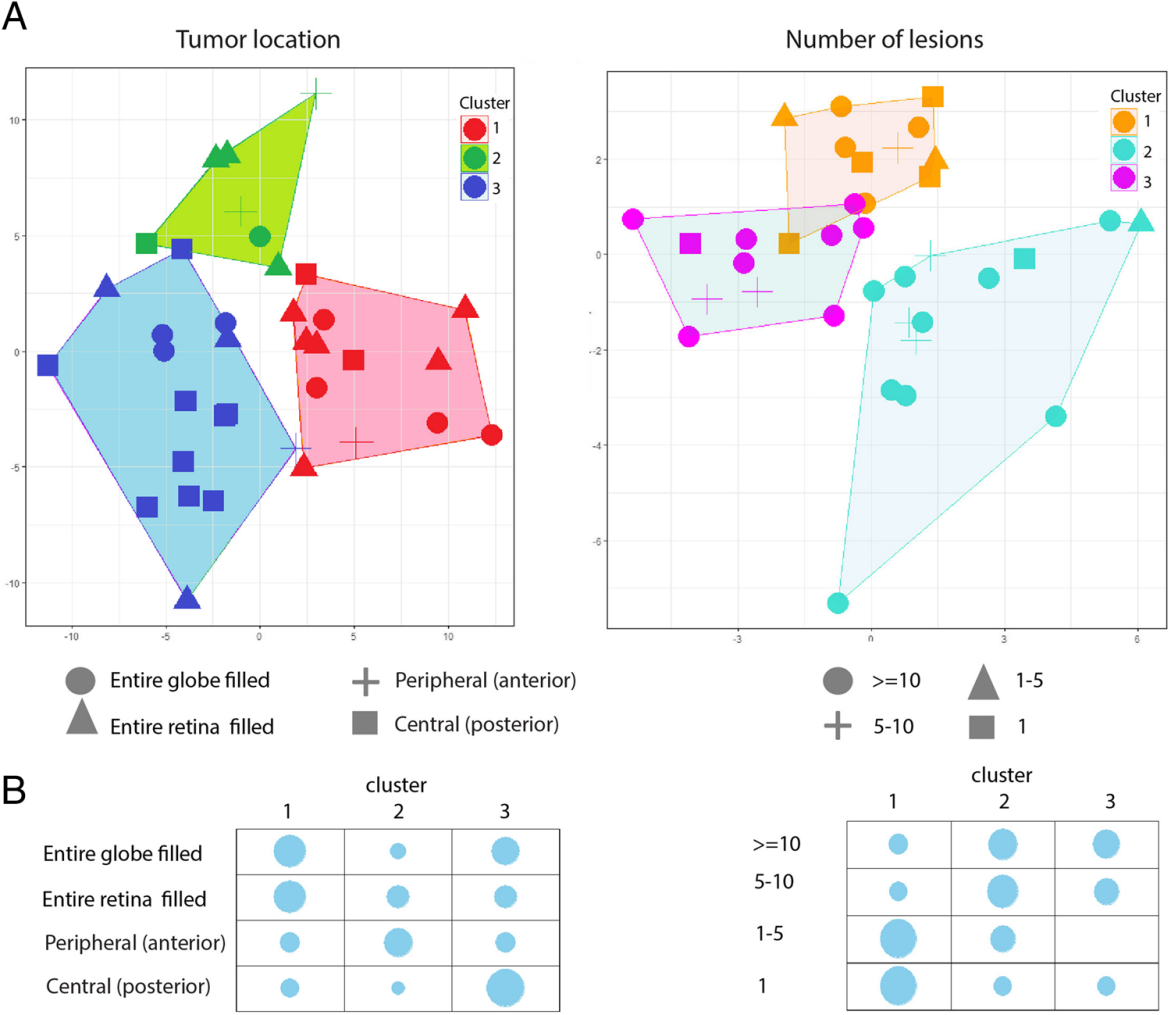
*K*-Means clustering analysis of the differentially expressed genes among the MRI features based on the genes previously identified in the test cohort, but now according to their gene expression in the validation cohort. Clustering for MRI features tumor location (left) and number of lesions (right). **A** Principal component analysis (PCA) plots overlaid and partitioned at arbitrary cluster number 3 (*k* = 3), using the *K*-means method. Colors signify the cluster where the samples reside in. As it shows for tumor location differentially expressed genes, posterior tumors tend to predominantly converge in cluster 3 while tumors that fill the globe or retina are in cluster 1. For number of lesions, tumors harboring more than 5 lesions tend to be positioned in clusters 2 and 3, while tumors with less than 5 lesions are more predominantly located within cluster 1. **B** Bubble plots with bubble sizes indicating proportion values, illustrating the proportion of the samples with various features within each *K*-means cluster. Columns indicate the cluster number; rows indicate MRI features. Bubble sizes are adjusted based on the proportion values

This validation study partially validated the test study for differentially expressed genes based on MRI features. *K*-Means clustering (*k* = 3) based on the genes previously identified but with their expression levels in the current cohort again revealed clustering of imaging traits (Fig. 4). When clustering was based on differentially expressed genes for tumor location in the test set (*n* = 355), tumors located in entire globe or retina congregated in the validation set (cluster 1). Similarly, tumors central/posterior in the retina congregated in cluster 3. For the differentially expressed genes based on the number of lesions in the test set (*n* = 70), there was a close relationship between samples harboring 1 and 1–5 lesions in the validation set (cluster 1), while cluster 3 almost exclusively included tumors with 5–10 lesions or > 10 lesions. Regarding the other two imaging features associated with many differentially expressed genes in the previous study—subretinal and vitreous seeding—no distinctive clustering pattern was found in the current study (Appendix Fig. 2). Gene ontology enrichment for differentially expressed genes per imaging feature identified in the current validation study did not show overlap with finding from the test study (Appendix Table 2). Additionally, previous imaging features associated with *SERTAD3* and *KAL1* genes were not validated in this study.

## Discussion

Radiogenomics is an emerging field that can aid in non-invasive genotyping, contributing to diagnostic, prognostic, and targeted treatment approaches. This study partially validated previous findings of radiogenomics in retinoblastoma using MRI and gene expression profiles. Validated results include the association between low expression of the photoreceptorness gene expression profile with MRI features of multiple tumors, a tumor encompassing the entire retina or entire globe, and a diffuse growth pattern. These results suggest that radiogenomics in retinoblastoma can be used among different centers, different MRI scanners, and different gene expression platforms.

MRI is incorporated in standard-of-care for retinoblastoma evaluation in high-income countries. It supports ophthalmologic diagnosis, assesses extent of disease, and screens the central nervous system for associated pathology. MRI quality has drastically improved over the past two decades, resulting in higher quality data for the prediction of parameters such as gene expression profiles (radiogenomics). Developing such techniques are vital, as tissue biopsies cannot be performed in retinoblastoma. Alternative minimally invasive methods are also currently being developed, with genotyping using liquid biopsy from the aqueous humor showing great potential as well [27].

Parallel with the advances in imaging, recent developments in genomics analysis have enhanced knowledge on the retinoblastoma oncogenic process and bio/prognostic markers, as well as its molecular subtypes. The identification of retinoblastoma major genomic components is pivotal in the development of tailored targeted therapies and prognosis predictors [28]. The photoreceptorness trait presents one of such genomic features. It signifies the averaged expression of a set of visual perception–associated genes that can divide retinoblastoma into two major subgroups, interacting in continuous fashion [7].

The three imaging features that were repeatedly correlated to low photoreceptorness were advanced stage imaging features, which is concordant with previous reports that low photoreceptorness corresponds to tumor progression and dedifferentiation [7]. These imaging findings (multiple tumors, a tumor encompassing the entire retina or entire globe, and a diffuse growth pattern) were now validated in a heterogeneous cohort using different gene expression analysis platforms. Notably, the diffuse growth was an imaging finding of tumor growing along the retina (versus endofytic or exofytic growth), which does not directly match the diffuse infiltrative retinoblastoma described on fundoscopy [29]. As a second approach next to examining photoreceptorness, genes identified as differentially expressed among MRI features in the test set were again evaluated. Clustering analysis examining the relationships between samples based on expression of these genes showed again congregation of cases with similar imaging outcomes for two imaging features. The results suggest that multiple approaches in radiogenomics can be (partly) validated.

There were, however, important discrepancies between the outcomes of the test study and the validation study. An association with low photoreceptorness was found for larger eye size, heterogeneous tumor, and presence of subretinal seeding only in the test set (not validated) and irregular tumor margins only in the validation set. Again a proportion of retinoblastoma showed diffuse-growing, plaque-shaped multifocal tumors, but the association with *KAL1* and *SERTAD3* was not validated. Similarly, gene ontology enrichment of differentially expressed genes resulted in different pathways in the test and validation set. Potentially, these discrepant findings reflect false discoveries in the test study or differences between study cohorts regarding contrast design or sample size. Furthermore, low inter-reader agreement may be an important factor in, for example, for eye size, showing as well low inter-reader agreement as discrepant findings for its association with photoreceptorness. Technical differences and imaging quality may also have influenced results. The higher field strength of the MRI systems included in the validation did not lead to an increased amount of radiogenomic associations. Nevertheless, it seems rational to use the highest quality MRI available for identifying associations with high-throughput data such as gene expression. The discrepancies between tests and validation analyses emphasize the importance of validation studies for radiogenomics.

Although some radiogenomic findings for photoreceptorness were partially validated, no targeted therapies are available for this molecular trait. However, a differential ex vivo chemotherapy sensitivity was found for photoreceptorness gene expression, implying its potential treatment relevance [7]. More importantly, the ability of cross-center validation of some of the radiogenomic results in this study suggests the potential for capturing molecular features. Parallel, potential treatment targets are being investigated for retinoblastoma [28, 30]. For example, the rare and aggressive *MYCN*-amplified *RB1-*wildtype subtype retinoblastoma is more therapy resistant to traditional chemotherapies, but equally chemo-sensitive to pevonedistat (a neddylation inhibitor), which is being developed as treatment for neuroblastoma [28, 31–33]. Other potential treatment approaches include inhibiting DNA repair protein RAD51 resulting in in vitro and in vivo antitumor effects [34]. Parallel advancement of radiogenomics identifying the molecular traits on which the treatment is targeted may aid patient selection and facilitate moving effectively from bench-to-bedside. Although no such clinical predictors were presented yet, validation remains a vital step towards clinical implementation. Radiogenomics may be similarly valuable for other intra-ocular tumors, such as uveal melanoma for which targeted therapies for molecular subtypes are being investigated [35].

An important impediment in moving towards clinical application of radiogenomic analysis is the unsatisfactory inter-reader agreement for imaging features. The recent advantages of extracting features from MR images using artificial intelligence (quantitative imaging or radiomics) may enable more reliable subtype prediction [9]. Automated tumor segmentation/delineation [36] and radiomics prediction models for post-laminar optic nerve invasion showed promising early-stage results [37].

This study has several limitations. Although the use of different imaging and gene expression techniques in different centers indicated the robustness of the validated results, it may have diminished statistical power. Sample size was limited due to scarcity of datasets of retinoblastoma with gene expression profile and MRI data. Another important limitation is the varying, and sometimes low, inter-reader agreement found in this study, which could partly also be explained by use of a wider range of MRI assessment years including older low-quality images.

In conclusion, MRI radiogenomic associations were partly validated in an independent cohort, indicating the importance of validation studies in radiogenomics. The validated results indicate the potential of radiogenomics to estimate retinoblastoma molecular subtypes and to guide future targeted treatment.

### Supplementary Information

Below is the link to the electronic supplementary material.

Supplementary file1 (PDF 393 KB)

## Abbreviations

GLM: Generalized linear model
hg38: Human Reference Genome 38
MRI: Magnetic resonance imaging
PCA: Principal component analysis
RMA: Robust multi-chip average
SIAC: Selective intra-arterial chemotherapy
TMM: Trimmed mean of *M*-value

## Declarations

### Guarantor

Pim de Graaf had full access to all the data in the study and take responsibility for the integrity of the data and the accuracy of the data analysis.

### Statistics and biometry

Two of the authors, K. Roohollahi and M. de Jong, have significant statistical expertise.

### Study subjects or cohorts overlap

Seventeen patients were previously reported in a study on genetic markers for high-risk retinoblastoma without imaging [16], while the current study reports on associations between MRI features and gene expression profiles.

16 Hudson LE, Mendoza P, Hudson WH et al (2018) Distinct gene expression profiles define anaplastic grade in retinoblastoma. The American Journal of Pathology 188:2328–2338.

Some study subjects or cohorts have not been previously reported.

### Methodology

- Retrospective
- Multicenter cohort study
