# Supplementary material for: Correlation of gene expression with magnetic resonance imaging features of retinoblastoma: a multi-center radiogenomics validation study
Source: Eur Radiol. 2023 Aug 24;34(2):863–72. doi: 10.1007/s00330-023-10054-y (PMC10853293; doi:10.1007/s00330-023-10054-y)

Appendix Table 1: Cohen's Kappa for interreader agreement of imaging features

MRI feature	**Cohen's Kappa**
Number of lesions	0.38
Growth pattern	0.55
Tumor location	0.29
Eye size	0.18
Enhancement choroid beneath tumor	0.50
Tumor homogeneity	0.37
Dominating shape of largest tumor lesion	0.42
Irregular/ill-defined tumor margins	0.48
Calcifications	0.29
Compactness of the entire mass	0.23
Enhancement anterior eye segment	0.74
Proportion contrast enhancing tumor (CET)	0.40
Proportion necrosis	0.36
Retinal detachment	0.68
Shallowness of the anterior eye chamber	0.68
Subretinal composition	0.60
Subretinal seeding	0.42
Vitreous hemorrhage	0
Vitreous seeding	0.5

Appendix Table 2: Functional annotation of differentially expressed genes between MRI features

MRI Feature	Site	Comparison	Differential expression	No.DEGs	Functional annotation	FDR
Tumor location	Amsterdam	Entire globe filled vs. Greater part of tumor behind	Upregulated	220	Organelle fission	1.8E-7
					Cell cycle process	2.5E-7
					DNA repair	2.1E-7
			Downregulated	456	Sensory organ development	4.4E-11
					Cell adhesion	1.5E-11
					Visual perception	1.5E-9
Tumor location	Amsterdam	Entire retina filled vs. Greater part of tumor behind	Upregulated	111	Nuclear division	1.6E-18
					Organelle fission	6.6E-18
					Cell cycle process	1.8E-17
			Downregulated	677	Sensory organ development	2.8E-22
					Cell adhesion	4.07E-19
					Visual perception	3.01E-17
					Regulation of cell motility	2.09E-16
Tumor location	Atlanta	Entire retina filled vs. Greater part of tumor behind	Downregulated	25	Visual perception	2.37E-7
Virtuous seeding	Amsterdam	Outside 3mm range vs. inside 3mm range	Upregulated	18	Extracellular matrix organization	1.99E-3

Supplementary Materials

Appendix Figure 1

Association of imaging features with photoreceptorness gene expression. Plaque-shaped tumors (top) and tumor with irregular borders (bottom) showed lower photoreceptorness. Results were only found in the current cohort (validation set) and not in the previous cohort (test set).

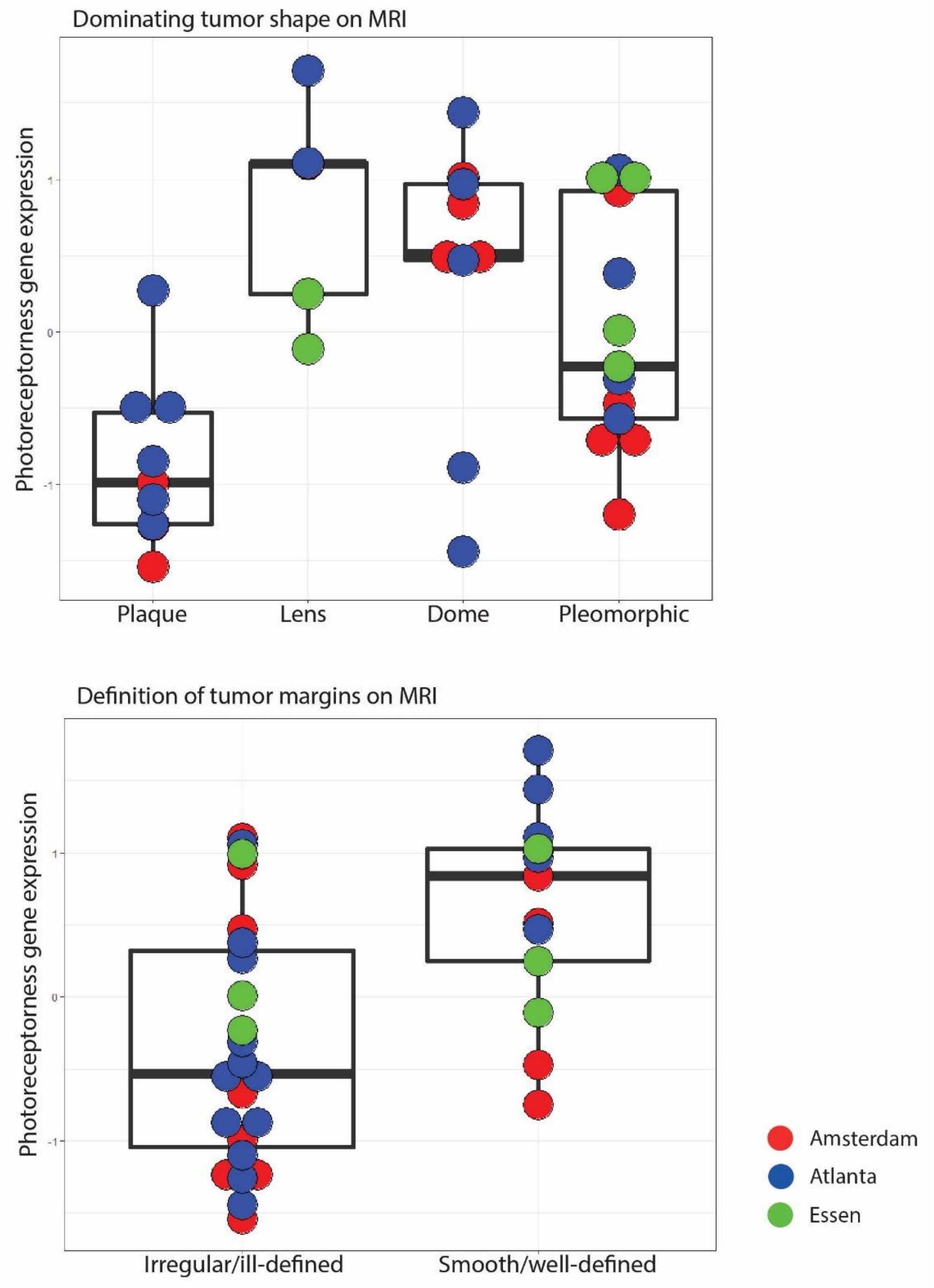

Appendix Figure 2

K-means clustering analysis of the differentially expressed genes amongst the MRI features based on the genes previously identified in the test cohort, but according to their expression in the validation cohort. Clustering for subretinal seeding (top) and vitreous seeding (bottom).

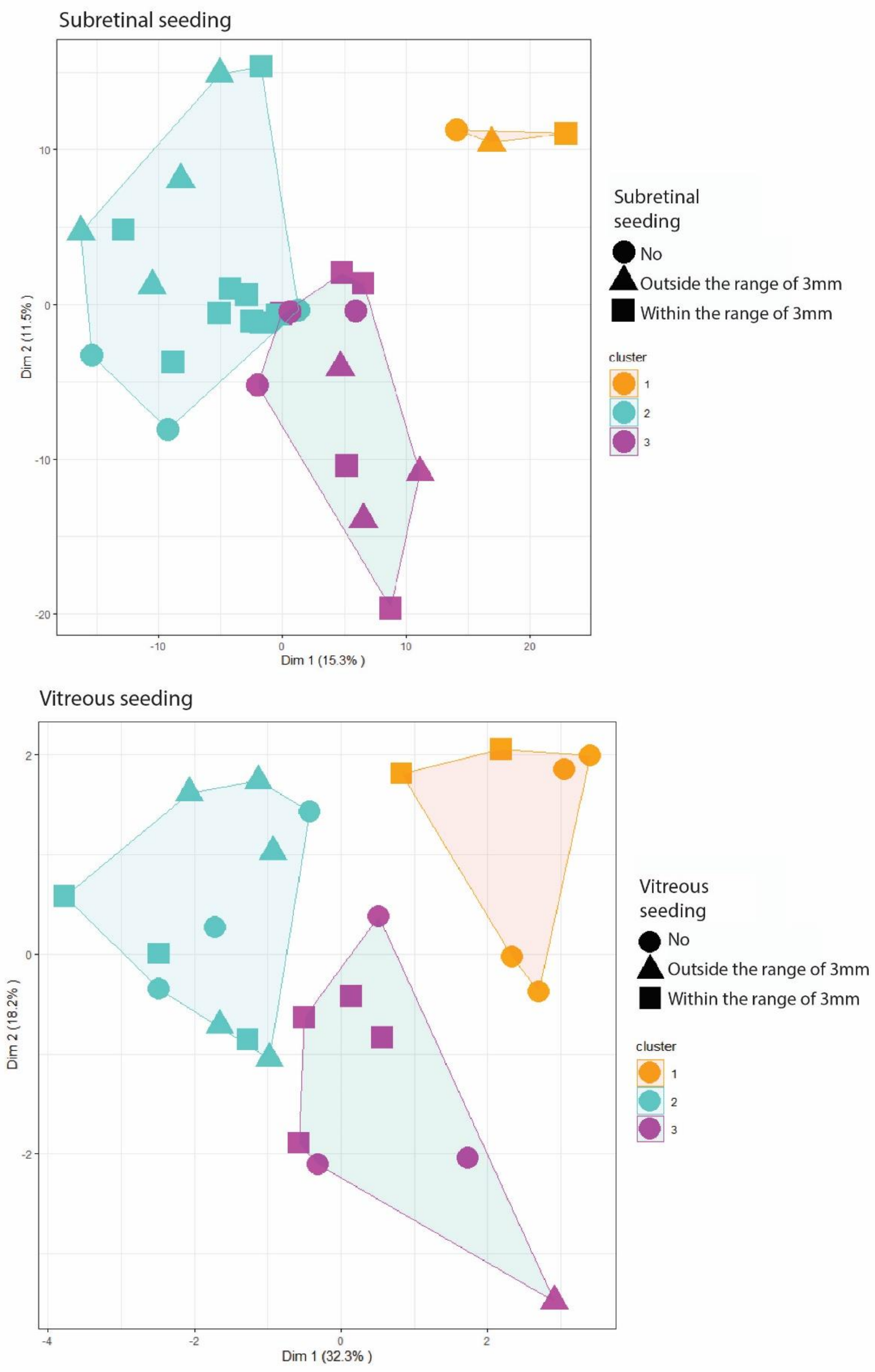

Supplement: Supplementary file 1 — Supplementary file1 (PDF 393 KB) [file 330_2023_10054_MOESM1_ESM.pdf]
